# Evaluating the Push-Up Challenge: Impacts on Mental Health, Help-Seeking and Exercise Behaviours in Australia

**DOI:** 10.1007/s10935-025-00872-9

**Published:** 2025-08-20

**Authors:** Nina Logan, Amy Morgan, Anna Ross, Ellie Tsiamis, Nicola Reavley

**Affiliations:** 1 Melbourne School of Population and Global Health, Centre for Mental Health and Community Wellbeing), University of Melbourne, Melbourne, VIC Australia; 2 Melbourne School of Population and Global Health, Centre for Health Equity), University of Melbourne, Melbourne, VIC Australia

**Keywords:** Mental health promotion, Physical activity, Help-seeking behaviours, Mental health literacy, Social connectedness, Population-based intervention

## Abstract

The Push-Up Challenge is an Australian health promotion event combining mental health awareness with an exercise goal. This study aimed to evaluate its impact on participants’ resilience, wellbeing and mental health, physical activity, social connectedness, and mental health literacy. Using a pre-post design, we analysed survey data across three timepoints: pre-event (*N* = 29,069), two weeks post-event (*N* = 9,970), and three months post-event (*N* = 4,346). Outcomes included resilience, depression and anxiety symptoms, wellbeing, help-seeking behaviours, self-care activities, and physical activity levels. Survey respondents were mostly male (64.8%) with a mean age of 35.68 years (SD = 12.8). Mixed-effects models were used to assess change over time, adjusting for factors associated with missingness. At three-month follow-up, results showed very small significant reductions in depression (d = -0.09, *p* < 0.001) and anxiety symptoms (d = -0.09, *p* < 0.001), and small improvements in positive wellbeing (d = 0.15, *p* < 0.001). There were no significant changes in resilience, physical activity, social connection, or odds of experiencing a mental health problem. Participants were more likely to seek help for mental health problems (OR = 2.05 [1.31, 3.19]) and adopt self-care strategies (OR = 3.79 [2.64, 5.45]). Physical activity levels increased significantly post-event (d = 0.10, *p* < 0.001) but were not maintained at follow-up. While improvements in mental health symptoms were small, this is consistent with similar population-level mental health promotion interventions. Findings suggest that The Push-Up Challenge shows promise as a multifaceted intervention combining exercise-based mental health promotion with mental health awareness and literacy components.

## Background

Improving mental health in Australia is a critical public health priority, with half of all Australians experiencing a mental illness at some time in their life (Australian Bureau of Statistics, 2020–2022). Each year, 22% of adults and 39% of young people (16–24 years) experience a mental illness (Australian Bureau of Statistics, 2020–2022), and in 2023 3,214 Australians died by suicide (Australian Bureau of Statistics, 2023). Mental illness and substance use disorders are the second leading cause of disease burden in Australia, accounting for 15% of the total (Australian Institute of Health and Welfare, 2024).

Population-level mental health promotion campaigns play a crucial role in addressing the burden of mental illness in Australia by targeting key determinants of mental health, such as mental health literacy, resilience, social connection, and self-care behaviours (Anwar-McHenry et al., 2012; Jorm et al., 2005). They also aim to help people recognise the signs and symptoms of mental health problems, seek appropriate support, and provide support to others in need. By fostering these changes both in those at risk of mental health problems and their broader social networks, mental health promotion campaigns can reduce the overall impact of mental illness on the population (Ross & Bassilios, 2019; Xu et al., 2018).

Such campaigns have been found to improve mental health literacy, help-seeking intentions, and attitudes towards people with mental illness, both in Australia and globally, but do not always improve actual help-seeking in the general public (Tam et al., 2024; Xu et al., 2018). This may be because observable behaviour change often requires larger study sample sizes and longer-term follow-up (Xu et al., 2018).

In Australia, two well-known mental health campaigns are *Beyond Blue*, which was found to improve public knowledge and beliefs about mental health (Jorm et al., 2005), and the *R U OK? Day* initiative, which was also associated with enhanced helping attitudes, increased confidence to offer support, and engagement in supportive behaviours (Ross & Bassilios, 2019). Globally, exposure to mental health campaigns has also been linked to improved mental health outcomes, including reduced stress and depressive or anxiety symptoms, as well as lower rates of suicidal thinking (Tam et al., 2024). These benefits are often correlated with other outcomes such as higher rates of formal help-seeking (Xu et al., 2018), increasing social connection and support (Anwar-McHenry et al., 2012), and modelling coping behaviours (Niederkrotenthaler & Till, 2020).

Physical activity is also an important mental health determinant to target through health promotion. A growing body of evidence indicates that physical activity can reduce symptoms of stress, prevent and manage mental health problems, and improve mood, self-esteem and sleep quality (Chekroud et al., 2018; Mahindru et al., 2023; Marinelli et al., 2024). Social support can further facilitates the uptake and maintenance of physical activity through encouragement, sharing of resources, and companionship (Golaszewski et al., 2022). However, evaluations of community-based campaigns aimed at increasing physical activity have yielded mixed findings, with some, such as *10*,*000 Steps Rockhampton*, reporting positive effects (Brown et al., 2006), and others, such as *This Girl Can*, showing limited behavioural impact (Bauman et al., 2023). Brown et al. (2006) attributed the success of their campaign to the involvement of the whole community in its planning and high awareness of its messaging. Conversely Bauman et al. (2023) described campaign awareness as average for a media campaign and did not describe the ways that community was involved.

The Push-Up Challenge is a national Australian health promotion event that integrates mental health awareness with a physical fitness challenge. Delivered by the *Push for Better Foundation*, the Push-Up Challenge tasks participants to complete 3,249 push-ups over a 24-day period; reflecting the number of Australians who died by suicide in 2022. Each daily push-up target is linked to a mental health statistic, accompanied by educational content designed to raise awareness, promote help-seeking and encourage self-care. Since its launch in 2017, the Push-Up Challenge has engaged over 700,000 participants. Individuals receive their daily target and facts via an app, take part either solo or in teams, and may substitute push-ups for other exercises. Participants are also encouraged to raise funds for mental health causes and organisations, and share progress and mental health facts on social media to foster community engagement and conversation around mental health.

There has been no external evaluation of The Push-Up Challenge to date. Therefore, the aim of this study was to evaluate the impact of The Push-Up Challenge on participants’ resilience, wellbeing and mental health, physical activity, social connectedness and mental health literacy.

## Methods

### Aims

This evaluation aimed to assess whether participation in the Push-Up Challenge improved resilience, wellbeing and mental health, exercise habits, and related behaviours. Primary outcomes were changes in physical activity, wellbeing, and resilience. Secondary outcomes were symptoms of depression and anxiety, social connection, confidence in providing mental health support, attitudes towards physical activity, help-seeking intentions, and self-care. It was hypothesised that participants would demonstrate significant improvements in the primary and secondary outcomes following participation. Exploratory analyses examined whether changes varied by either baseline mental health symptom severity or completion of the push-up target, with the hypothesis that greater improvements would be observed among both those with higher initial symptom severity and those who achieved their exercise goal.

### Design

A pre-post survey design was used, with data collected at three timepoints; pre-event, two weeks post-event and three months post-event. To reduce participant burden, a brief survey assessed primary outcomes while optional additional survey questions captured secondary outcomes.

Demographic information (age, gender) and Challenge participation details (full or half push-up goal) were collected through the app. Participants used the app to log their daily push-up total. Data were provided to the research team by the *Push For Better Foundation* in anonymised format and the research team did not have access to any identifying information.

### Participant Eligibility and Recruitment

All participants aged 16 years and older in the 2024 Push-Up Challenge event were eligible. Study participation was voluntary. Recruitment occurred via email and the participant app which connected them to the Plain Language Statement, consent form, and surveys. Due to many participants uninstalling the app after the event, the three-month follow-up survey was also distributed via SMS.

### Intervention

Participants were tasked with completing 3,249 push-ups (or an equivalent exercise) over a month, with daily targets linked to mental health statistics. Daily mental health facts were provided to raise awareness, encourage self-care, and promote help-seeking behaviours. Content was delivered digitally via the participant app and email communications. Participants could take part individually or in self-organised teams. The intervention was delivered by the *Push for Better Foundation*, a not-for-profit organisation. The Push-Up Challenge was conducted in participants’ homes, workplaces, and community spaces, as it was a remote, app-based event. Participants received daily mental health facts and exercise targets across the month. The Push-Up Challenge occurred between 5 and 28 June 2024. To encourage participation, the app included daily reminders and progress tracking. Participants could also share progress on social media and fundraise for mental health organisations.

### Primary Outcomes

#### Wellbeing

Wellbeing was measured using the Short Warwick-Edinburgh Mental Well-being Scale (SWEMWBS; Tennant et al., 2007), a 7-item scale designed to assess mental wellbeing. The SWEMWBS assesses positive aspects of mental health, such as feelings of optimism, resilience, and social functioning, over the past two weeks. Higher scores indicate greater mental wellbeing (range = 7–35). The scale demonstrated good internal consistency, with McDonald’s omega = 0.86.

#### Resilience

Resilience was measured using the CD-RISC2, a two-item version of the Connor-Davidson Resilience Scale (Vaishnavi et al., 2007). The CD-RISC2 assesses resilience by asking about participants’ ability to bounce back from stress and to adapt to change. Higher scores indicate greater resilience. The scale demonstrated lower internal consistency, with Spearman-Brown coefficient = 0.64.

#### Physical Activity Behaviours

The Godin Leisure-Time Exercise Questionnaire (GLTEQ; Godin et al., 1985) was used to assess physical activity levels. Participants reported the frequency of mild, moderate, and vigorous activities in a week, scored as mild × 3, moderate × 5, and vigorous × 9. Scores were summed, with higher totals indicating greater activity levels (range = 0–199). The scale demonstrated acceptable internal consistency, with McDonald’s omega = 0.73.

### Secondary Outcomes

#### Mental Health Symptoms

Depression symptoms were measured using the Patient Health Questionnaire-2 (PHQ-2; Löwe et al., 2005), a two-item screening tool for depression derived from the Patient Health Questionnaire (PHQ-9; Kroenke et al., 2001). The PHQ-2 assesses the frequency of depressive symptoms over the prior two weeks. Higher scores indicate greater severity of depression symptoms (range = 0–6). The scale demonstrated lower internal consistency, with Spearman-Brown coefficient = 0.64.

Anxiety symptoms were measured using the General Anxiety Disorder 2-item (GAD-2), a two-item screening tool for generalised anxiety, adapted from the Generalised Anxiety Disorder Assessment (GAD-7; Spitzer et al., 2006). The GAD-2 assesses the frequency of anxiety symptoms over the past two weeks. Higher scores indicate greater severity of anxiety symptoms (range = 0–6). The scale demonstrated acceptable internal consistency, with Spearman-Brown coefficient = 0.79.

#### Social Connection

This was measured using the Acceptance/Inclusion subscale of the General Belongingness Scale (AI-GBS; Malone et al., 2012). This subscale assesses the extent to which individuals feel accepted and included by others in their social environment. Higher scores indicate a greater sense of acceptance and inclusion (range 0–8). The scale demonstrated excellent internal consistency, with McDonald’s omega = 0.93.

#### Mental Health Behaviours

Participants were asked if they had experienced a mental health problem in the past four weeks; “Over the last 4 weeks, have you yourself had any sort of mental health problem?”. Those who responded “yes” were asked about subsequent help-seeking actions (health professional; friend or family; religious leader; medication; digital mental health program; online mental health support group or forum). All participants reported on utilisation of self-care activities over the past four weeks (social connection; exercise; diet; nature; screen time; sleep habits; connection to animals; kindness to others) and rated their confidence in supporting someone with a mental health problem on a 5-point scale ranging from 1 = “Not confident at all” to 5 = “Completely confident”. They were also asked if they knew someone who had developed or experienced a worsening mental health issue or crisis in the past four weeks. If “Yes”, they indicated whether they had taken action to support them (“Yes” or “No”).

#### Physical Activity Attitudes

Attitudes towards physical activity were assessed with four items; “Physical activity is (1) easy, (2) relaxing, (3) enjoyable, and (4) healthy.” Participants rated agreement on a 5-point Likert scale ranging from 1 = Strongly disagree to 5 = Strongly agree. Responses were summed to produce an overall score, with higher scores indicating more positive attitudes towards physical activity. The scale demonstrated acceptable internal consistency, with McDonald’s omega = 0.77.

### Analysis

An intention-to-treat approach was used, including all participants who completed at least one survey. Mixed-effects models were used to analyse changes over time. This method is appropriate for longitudinal data with substantial attrition rates and involved the use of all available data under the assumption that data were missing at random (MAR), modelling both within-subject correlations and individual variability. Time and variables associated with missingness were included as fixed effects to adjust for bias due to dropout and meet the MAR assumption. To account for individual differences in pre-event outcome scores and within-subject correlations, a random intercept was included for each participant. Logistic mixed-effects models used multi-dimensional adaptive quadrature with 30 integration points. Depression, anxiety, resilience and physical activity attitude variables were Box-Cox transformed to improve distribution of residuals and heteroscedasticity.

Post-hoc subgroup analyses were conducted for participants with clinical symptoms of depression and anxiety (≥ 3) using interaction terms in the adjusted models for these variables. A similar analysis was also performed to investigate changes to depression and anxiety symptom severity between participants who did and did not complete the required number of push-ups. Such analyses were exploratory and only used available data.

McDonald’s omega was used to assess the internal consistency of the scalar measures pre-event for scales with more than two items. The Spearman-Brown coefficient was used for two-item scales.

Effect sizes (Cohen’s d) were calculated by dividing the estimated mean difference by the pre-event standard deviation. The following thresholds were applied to describe the magnitude of effects: negligible (< 0.05), very small (0.05–0.19), small (0.20–0.49), medium (0.50–0.79), large (≥ 0.80), and very large (≥ 1.20).

Data processing, cleaning, matching, and analysis were completed using RStudio and Stata/SE 18. Significance was set at *p* < 0.01 for multiple testing and *p* < 0.05 for missingness.

### Ethics and Reporting Information

The study was approved by the University of Melbourne Human Ethics Committee (2024-29596-53636-3). The reporting of this study follows the TREND statement guidelines for transparent reporting of evaluations of nonrandomised designs (Des Jarlais et al., 2004), with the completed TREND checklist included in the supplementary materials.

## Results

### Survey Participant Flow and Numbers Analysed

**Fig. 1:**
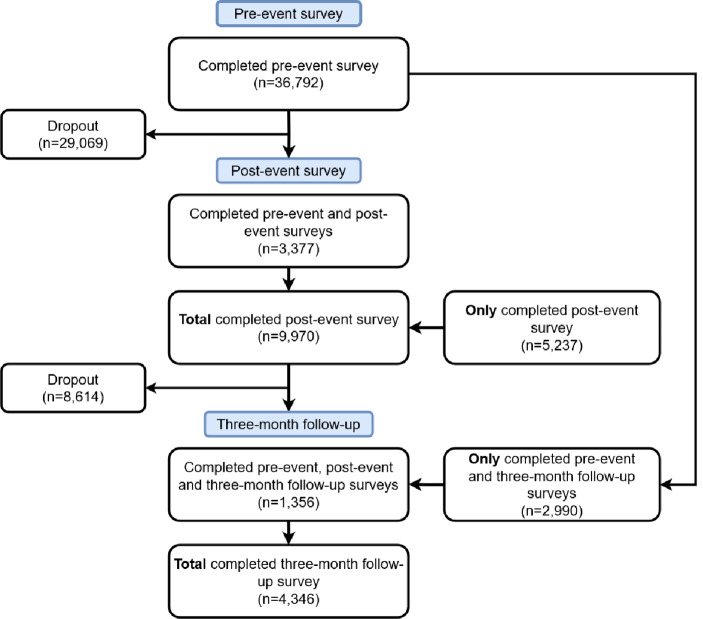
Participant flow diagram of survey completion and dropout rates across three stages: pre-event stage, 36,792 participants completed the survey, then 29,069 dropped out; two weeks post-event stage, 9,970 completed the survey, including 3,377 who also did the pre-event survey, then 8,614 dropped out; three months post-event stage, 4,346 completed the survey, including 1,356 who completed all three surveys

The flow diagram for the number of participants at each timepoint is given in Fig. 1. A total of 42,049 unique participants took part in the study. Most only completed the T1 survey (*n* = 29,069, 69%) or T2 survey (*n* = 5,237, 12%). Less than a fifth (*n* = 7,723, 18%) completed surveys at more than one timepoint with only 3% (*n* = 1,356) completing all three surveys.

### Characteristics and Intervention Participation

The mean age of participants who completed the pre-event survey at baseline was 35.8 (SD = 12.8) and 64.8% were male. Most of these participants (85.4%) aimed to complete the full push-up target (3,249 push-ups). 39% of those aiming for the full target and 24.0% of those aiming to complete the half target (1,625 push-ups) completed this goal by the end of the Push-Up Challenge. Among all pre-event survey respondents, the mean number of push-ups completed over the event duration was 1,972 (SD = 1,229.1). Compared to the total event cohort, pre-event survey respondents were older (M = 35.8 vs. 31.7 years) and slightly less likely to be male (64.8% vs. 67.2%). A greater proportion aimed for the full push-up target, compared to the total event cohort (85.4% vs. 80.0%). Participant demographic and engagement characteristics are presented in Table 1, stratified by survey response cohort; the subgroup of respondents that completed a questionnaire at each survey timepoint.

**Table 1:** Participant demographic and event engagement characteristics by survey response cohort

Attributes	Total event cohort (*n* = 237,340)	Pre-event survey respondents		Two weeks post-event survey respondents		Three months post-event survey respondents	
		Brief (*n* = 36,792)	Additional questions (*n* = 15,691)	Brief (*n* = 9,970)	Additional questions (*n* = 2,000)	Brief (*n* = 4,346)	Additional questions (*n* = 2,470)
Age, M (SD)	31.7 (13.8)	35.8 (12.8)	37.9 (12.3)	36.8 (14.3)	40.5 (13.3)	38 (13.8)	40 (13.0)
Gender, n (%)							
Male	156,976 (67.2)	23,548 (64.8)	9,535 (61.5)	6,631 (67.3)	1,235 (62.2)	2,842 (66.4)	1,551 (63.6)
Female	75,740 (32.0)	12,543 (34.5)	5,866 (37.8)	3,156 (32.0)	740 (37.3)	1,412 (33.0)	870 (35.7)
Nonbinary	1,893 (0.8)	241 (0.07)	114 (0.07)	71 (0.07)	9 (0.05)	27 (0.06)	17 (0.07)
Push-up target, n (%)							
Full	181,903 (80.0)	31,419 (85.4)	13,479 (85.9)	8,891 (89.2)	1,792 (89.6)	3,861 (88.8)	2195 (88.9)
Half	45,545 (20.0)	5,370 (14.6)	2,211 (14.1)	1,079 (10.8)	208 (10.4)	485 (11.2)	275 (11.1)
Target completed by end of event, n (%)							
Full	N/A	12,252 (39.0)	5,628 (41.8)	6,926 (77.9)	1,339 (74.8)	2,347 (60.7)	1,364 (62.1)
Half	N/A	1,291 (24.0)	560 (25.3)	658 (61.0)	113 (54.3)	223 (46.0)	124 (45.1)
Number of push-ups recorded over duration of event, M (SD)	1,765.5 (1,257.5)	1,972.6 (1,229.1)	2,046.8 (1,220.0)	2,861.0 (764.9)	2,823.1 (802.7)	2,527.0 (1,025.7)	2,541.1 (1,021.5)

### Predictors of Missingness

Missingness was defined as completing only one survey. 34,306 participants who completed a brief survey only (81.7%) and 11,890 participants who also completed the optional additional questions (75.8%) were considered missing.

The following baseline variables were significantly associated with increased odds of missingness: having a half push-up target rather than a full push-up target, not completing the push-up target, being in an older age group, having more severe symptoms of depression, having more negative attitudes towards physical activity and experiencing a mental health problem in the past 4 weeks. Additionally, being in a younger age group and having lower wellbeing were also associated with increased odds of missingness for those who completed only the brief survey.

### Observed Outcomes in Survey

Scores at each timepoint for continuous outcomes and proportions for binary outcomes are presented in Table 2. In the pre-event survey, reported mean scores for wellbeing (M = 24.60, SD = 4.30) are similar to population norms (Tennant et al., 2007). Mean scores for resilience (M = 5.81, SD = 140) also fall within the average range of scores for the full CD-RISC scale (Vaishnavi et al., 2007).

Physical activity behaviour was also moderate pre-event (M = 65.65, SD = 36.57) with a mean equivalent of 196 minutes of moderate exercise per week (Godin et al., 1985), falling within the recommended range of exercise duration for Australian adults (150–500 min per week; Australian Government Department of Health and Aged Care, 2021).

**Table 2:** Observed means (standard deviations) for continuous outcomes and proportions with counts (%) for binary outcomes, assessed pre-event, two weeks post-event, and three months post-event

Outcome	Pre-event		Two weeks post-event		Three months post-event	
	M (SD)	N	M (SD)	N	M (SD)	N
Wellbeing	24.60 (4.30)	34,079	25.20 (4.37)	9,377	25.13 (4.27)	4,346
Resilience	5.81 (1.40)	34,779	5.94 (1.38)	9,540	5.63 (1.42)	3,957
Physical activity (behaviour)	65.65 (36.57)	27,475	70.79 (38.55)	9,622	65.53 (36.18)	4,346
***Secondary outcomes***						
Depression symptoms*	1.55 (1.42)	15,565	1.31 (1.39)	1,978	1.46 (1.47)	2,470
Anxiety symptoms*	1.71 (1.54)	11,742	1.40 (1.44)	1,980	1.46 (1.47)	2,470
Social connection*	23.07 (4.39)	15,375	23.49 (4.48)	1,968	23.22 (4.42)	2,470
Confidence providing mental health support*	3.53 (1.06)	15,221	3.56 (1.07)	1,964	3.50 (1.03)	2,470
Physical activity (attitudes)*	17.17 (2.65)	15,108	17.78 (2.39)	655	17.44 (2.44)	2,470
Experience of a mental health problem* n (%)	*N* = 15,358		*N* = 1,978		*N* = 2,470	
No	9,026 (58.8)		1,252 (63.3)		1,612 (65.3)	
Yes	5,651 (36.8)		647 (32.7)		759 (30.7)	
Prefer not to say	681 (4.4)		79 (4.0)		99 (4.0)	
Help-seeking behaviours* ^†^ * n (%)*	*N* = 5,651		*N* = 1,978		*N* = 2,470	
Any help-seeking behaviour	4,475 (79.2)		568 (87.8)		646 (85.11)	
Health or mental health professional	1,772 (31.4)		239 (36.9)		292 (38.5)	
Friend or family	3,751 (66.4)		458 (70.8)		539 (71.0)	
Spiritual or religious leader	225 (4.0)		27 (4.2)		24 (3.2)	
Took medication	1,295 (22.9)		190 (29.4)		187 (24.6)	
Digital mental health treatment program	306 (5.4)		46 (7.1)		60 (7.9)	
Digital mental health forum or chat room	133 (2.4)		27 (4.2)		22 (2.9)	
Self-care activities* n (%)	*N* = 15,691		*N* = 2,000		*N* = 2,470	
Any self-care activity	13,957 (89.0)		1,942 (97.1)		2,397 (97.0)	
Connected with a friend or family	10,588 (67.5)		1,468 (73.4)		1,808 (73.2)	
Increased physical activity	10,158 (64.7)		1,637 (81.9)		1,779 (72.0)	
Improved diet	6,714 (42.8)		886 (44.3)		1,184 (47.9)	
Time in nature	7,574 (48.3)		1,128 (56.4)		1,517 (61.4)	
Reduced screen time	3,780 (24.1)		571 (28.6)		710 (28.7)	
Improved sleep habits	4,661 (29.7)		644 (32.2)		801 (32.4)	
Time with a pet	6,525 (41.6)		1,027 (51.4)		1,121 (45.4)	
Act of kindness	6,835 (43.6)		1,074 (53.7)		1,106 (44.8)	

* Asked in additional questions. ^†^Asked only of participants who said they were experiencing a mental health problem

### Changes in Primary Outcomes Across Timepoints

Table 3 includes the changes in primary outcomes over timepoints.

#### Wellbeing

Participants showed significant improvement in wellbeing from pre-event to two weeks post-event (0.83 [0.66, 1.00]; *p* < 0.001) and to three months post-event (0.64 [0.47, 0.80]; *p* < 0.001). These changes were small at both timepoints.

#### Resilience

Participants showed a significant improvement in resilience from pre-event to two weeks post-event (0.10 [0.03, 0.16]; *p* < 0.001). The magnitude of this change was very small. There were no significant changes in resilience from pre-event to three months post-event (−0.05 [−0.12, −0.01]; *p* = 0.012).

#### Physical Activity Behaviours

Participants showed a significant improvement in leisure time exercise from pre-event to two weeks post-event (3.65 [2.17, 5.13]; *p* < 0.001). The magnitude of this change was very small. No significant change was found from pre-event to three months post-event (−0.61 [−2.07, 0.86]; *p* = 0.285).

### Changes in Secondary Outcomes Across Timepoints

Table 3 includes the changes in continuous secondary outcomes across timepoints. Table 4 presents the changes in binary secondary outcomes.

#### Symptoms of Depression and Anxiety

A significant reduction in the severity of depression symptoms was found from pre-event to two weeks post-event (−0.14 [−0.21, −0.07]; *p* < 0.001) and three months post-event (−0.13 [−0.19, −0.07]; *p* < 0.001). Changes in depression symptom severity were very small across post-event timepoints. A significant reduction was also found in anxiety symptoms severity from pre-event to two weeks post-event (−0.20 [−0.28, −0.13]; *p* < 0.001) and three months post-event (−0.15 [−0.22, −0.08]; *p* < 0.001). These changes were very small across post-event timepoints. 

#### Subgroup analysis: participants with anxiety or depression symptom severity above the clinical threshold

Changes in depression symptom severity over time differed significantly between participants with clinical symptoms of depression and the full sample. At two weeks post-event, this subgroup showed a greater reduction in depression symptom severity compared to the full sample (−1.39 [−1.52, −1.26]; *p* < 0.001). Similarly, at three months post-event, the reduction in depression symptom severity for the subgroup was larger than that of the full sample (−1.54 [−1.66, −1.42]; *p* < 0.001). The magnitude of the change in depression symptoms for this subgroup was large at both two weeks (d = −0.98 [−1.02, −0.94]) and three months post-event (d = −1.08 [−1.12, −1.05]).

The change in anxiety symptom severity over time also differed significantly between participants with clinical symptoms of generalised anxiety and the full sample. Two weeks post-event, the subgroup showed a greater reduction in anxiety symptom severity compared to the full sample (−1.01 [−1.16, −0.85]; *p* < 0.001). Similarly, three months post-event, the reduction in anxiety symptom severity for the subgroup was larger than that of the full sample (−1.32 [−1.46, −1.17]; *p* < 0.001). The magnitude of the change in anxiety symptoms for this subgroup was medium at two weeks post-event (d = −0.65 [−0.70, −0.61]) and large at three months post-event (d = −0.86 [−0.90, −0.81]).

#### Subgroup analysis: participants who completed the requisite number of push-ups

The change in depression symptom severity over time did not differ significantly between participants that completed their Push-Up Challenge goal (i.e., completed the required number of push-ups) and the full sample at either two weeks (−0.09 [−0.24, 0.06]; *p* = 0.123) or three months post-event (−0.01 [−0.13, 0.12]; *p* = 0.849).

Similarly, the change in anxiety symptom severity over time did not differ significantly between participants that completed their Push-Up Challenge goal and the full sample at either two weeks (0.12 [−0.05, 0.30]; *p* = 0.073) or three months post-event (0.03 [−0.11, 0.18]; *p* = 0.547).

#### Social Connection

Participants showed significant improvement in social connection from pre-event to two weeks post-event (0.33 [0.15, 0.50]; *p* < 0.001). The magnitude of this change was very small. There were no significant changes in social connection from pre-event to three months post-event (0.04 [−0.11, 0.20]; *p* = 0.470).

#### Experience of a Mental Health Problem

No significant change in the odds of experiencing a mental health problem were found two weeks post-event (OR 0.81 [0.64, 1.02]). Participants were at significantly lower odds of experiencing a mental health problem three months post-event (OR 0.65 [0.55, 0.76]) compared to their odds of experiencing a mental health problem pre-event.

#### Help-Seeking for a Mental Health Problem

Participants who did report experiencing a mental health problem were at significantly higher odds of reporting the utilisation of any form of help-seeking at both two weeks post-event (OR 2.92 [1.75, 4.90]) and three months post-event (OR 2.05 [1.31, 3.19]), compared to their odds of utilising any form of help-seeking pre-event.

From pre-event to two weeks post-event, significant increases were observed in the odds of taking a medication (OR 2.28 [1.20, 4.27]), and using a digital mental health support group, forum, or chat room (OR 2.28 [1.15, 5.93]). No significant changes were found in the other help-seeking options (health professional, friend or family, spiritual leader, digital mental health program). From pre-event to three months post-event, significant increases were observed in the odds speaking to a health professional (OR 1.78 [1.19, 2.66]), and to a family member or friend (OR 1.43 [1.05, 1.97]). No significant changes (*p* > 0.01) were found in the other help-seeking options (spiritual leader, medication, digital mental program, digital mental health forum).

#### Self-Care

Participants were at significantly higher odds of reporting that they engaged in any type of self-care activity two weeks (OR 3.72 [2.51, 5.53]) and three months post-event (OR 3.79 [2.64, 5.45]).

Two weeks post-event, participants were at significantly higher odds of connecting with a friend or family member (OR 1.25 [1.04, 1.50]), increasing exercise or physical activity (OR 2.54 [2.10, 3.07]), spending time in nature (OR 1.37 [1.15, 1.64]), reducing screen time (OR 1.44 [1.18, 1.75]), and spending time with a pet (OR 2.12 [1.67, 2.70]). No significant changes were found in the other self-care options (improved diet, improved sleep habits).

Three months post-event, participants were at significantly higher odds connecting with a friend or family member (OR 1.33 [1.12, 1.57]), increasing exercise or physical activity (OR 1.32 [1.13, 1.54]), improving diet (OR 1.29 [1.10, 1.51]), spending time in nature (OR 2.03 [1.72, 2.40]), reducing screen time (OR 1.45 [1.21, 1.73]), and spending time with a pet (OR 1.46 [1.18, 1.80]). No significant changes were found in the other self-care option (improved sleep habits).

#### Providing Mental Health Support

There were no significant changes found in participants’ confidence providing mental health support either from pre-event to two weeks post-event (0.03 [−0.02, 0.008]; *p* = 0.083) or to three months post-event (0.00 [−0.05, 0.04]; *p* = 0.959).

#### Physical Activity Attitudes

Participants showed a significant increase in positive attitudes towards physical activity from pre-event to two weeks post-event (0.24 [0.07, 0.41]; *p* < 0.001). The magnitude of this change was very small. No significant change was found from pre-event to three months post-event (0.04 [−0.05, 0.14]; *p* = 0.581).

**Table 3:** Adjusted changes in continuous outcomes over time (estimated mean difference and effect size from untransformed data provided for ease of interpretation; p-value based on variable transformation)

	Pre-event to two weeks post-event			Pre-event to three months post-event		
Outcome	Mean difference [99% CI]	*p*	d [99% CI]	Mean difference [99% CI]	*p*	d [99% CI]
***Primary outcomes***						
Wellbeing	0.83 [0.66, 1.00]	< 0.001	0.19 [0.15, 0.23]	0.64 [0.47, 0.80]	< 0.001	0.15 [0.11, 0.19]
Resilience*	0.10 [0.03, 0.16]	0.001	0.07 [0.02, 0.11]	−0.05 [−0.12, −0.01]	0.012	−0.04 [−0.08, 0.01]
Physical activity behaviours	3.65 [2.17, 5.13]	< 0.001	0.10 [0.06, 0.14]	−0.61 [−2.07, 0.86]	0.285	−0.02 [−0.06, 0.02]
***Secondary outcomes***						
Depression symptoms*	−0.14 [−0.21, −0.07]	< 0.001	−0.10 [−0.15, −0.05]	−0.13 [−0.19, −0.07]	< 0.001	−0.09 [−0.14, −0.05]
Anxiety symptoms*	−0.20 [0.28, −0.12]	< 0.001	−0.13 [−0.18, −0.08]	−0.15 [−0.22, 0.08]	< 0.001	−0.09 [−0.14, −0.05]
Social connection	0.33 [0.15, 0.50]	< 0.001	0.07 [0.03, 0.11]	0.04 [−0.11, 0.20]	0.470	0.01 [−0.03, 0.05]
Confidence providing mental health support	0.03 [−0.02, 0.08]	0.083	0.03 [−0.02, 0.08]	0.00 [−0.05, 0.04]	0.959	0.00 [−0.04, 0.04]
Physical activity attitudes*	0.24 [0.07, 0.41]	< 0.001	0.09 [0.03, 0.15]	0.04 [−0.05, 0.14]	0.581	0.02 [−0.02, 0.05]

* Variable transformed due to non-normal distribution of model residuals

**Table 4:** Adjusted changes in odds of binary outcomes over time

Secondary outcomes	Pre-event to two weeks post-event		Pre-event to three months post-event	
	OR [99% CI]	*p*	OR [99% CI]	*p*
Mental health problem in last four weeks	0.81 [0.64, 1.02]	0.019	0.65 [0.55, 0.76]	< 0.001
*Help-seeking*				
Any of the below	2.92 [1.75, 4.90]	< 0.001	2.05 [1.31, 3.19]	< 0.001
Health or mental health professional	1.33 [0.86, 2.05]	0.093	1.78 [1.19, 2.66]	< 0.001
Friend or family	1.32 [0.95, 1.86]	0.030	1.43 [1.05, 1.97]	0.003
Spiritual or religious leader	1.14 [0.48, 2.69]	0.691	0.60 [0.25, 1.45]	0.137
Took medication	2.28 [1.20, 4.27]	0.001	1.12 [0.61, 2.04]	0.626
Digital mental health treatment program	1.47 [0.80, 2.67]	0.103	1.67 [0.96, 2.87]	0.016
Digital mental health forum or chat room	2.61 [1.15, 5.93]	0.003	1.60 [0.70, 3.61]	0.141
*Self-care*				
Any of the below	3.72 [2.51, 5.53]	< 0.001	3.79 [2.64, 5.45]	< 0.001
Connected with a friend or family	1.25 [1.04, 1.50]	0.002	1.33 [1.12, 1.57]	< 0.001
Increased physical activity	2.54 [2.10, 3.07]	< 0.001	1.32 [1.13, 1.54]	< 0.001
Improved diet	0.99 [0.83, 1.18]	0.857	1.29 [1.10, 1.51]	< 0.001
Time in nature	1.37 [1.15, 1.64]	< 0.001	2.03 [1.72, 2.40]	< 0.001
Reduced screen time	1.44 [1.18, 1.75]	< 0.001	1.45 [1.21, 1.73]	< 0.001
Improved sleep habits	1.15 [0.96, 1.37]	0.045	1.17 [1.00, 1.37]	0.012
Time with a pet	2.12 [1.67, 2.70]	< 0.001	1.46 [1.18, 1.80]	< 0.001

## Discussion

This study aimed to evaluate the impact of the Push-Up Challenge, a community-based physical activity and mental health awareness intervention. Significant short-term improvements were found in resilience, social connection, physical activity, and help-seeking behaviours, while sustained improvements persisted in mental health symptoms, wellbeing, help-seeking and self-care at the three-month follow-up. The small effect sizes were consistent with findings from similar mental health promotion interventions. Importantly, small changes at the population level can contribute to meaningful public health benefits by shifting the distribution of mental health distress toward improved wellbeing when scaled across large groups (Rose et al., 2008).

### Mental Health and Wellbeing

Analysis revealed significant reductions in depression and anxiety symptom severity, as well as significant improvement in wellbeing, from pre-event to post-event and at three-month follow-up. However, effect sizes for reductions in depression and anxiety symptoms were very small to negligible, while the improvement in wellbeing demonstrated a small effect size, suggesting a relatively greater impact on positive mental health than symptom severity. Notably, there was no significant change in the odds of participants reporting that they experienced a mental health problem immediately after the event, but a significant reduction in these odds was observed at the three-month follow-up. Similar small effects on mental health symptoms have been reported in other exercise-based mental health promotion interventions for the general population across diverse age groups (Pascoe et al., 2020; Rosenbaum & Sherrington, 2011). These small effects may be attributable to the sample reporting relatively low pre-event mental health symptom severity, offering limited capacity for reduction. However, while positive mental health impacts on the overall sample may appear modest, they highlight more significant potential for population-level benefits as even minor improvements in a generally healthy population can aggregate to markedly reduce the overall impact of mental health problems (Rose et al., 2008).

Post-hoc subgroup analyses provided further insight into the potential benefits of the Push-Up Challenge, showing that participants with baseline depression or anxiety scores exceeding the clinical threshold experienced greater reductions in these symptoms than the overall sample. While, this pattern aligns with evidence that exercise-based mental health interventions often yield more substantial benefits for individuals with clinical symptoms (Kvam et al., 2016; Stubbs et al., 2017), these findings should be interpreted cautiously. The larger improvements may simply be due to a greater scope for change among those with higher baseline scores.

Additional analyses did not find any difference in improvements in mental health symptoms between those who did and did not complete requisite number of push-ups during the Push-Up Challenge event. Other elements of the event may be associated with these mental health improvements, with participants in previous exercise-based mental health promotion campaigns have described how engaging in exercise itself, regardless of goal achievement, promoted positive affect through improved physiological feelings and mood. They also described how this was augmented by exercising outdoors, or with other people (Wheatley et al., 2021).

### Exercise Behaviours and Attitudes

Participation in the Push-Up Challenge was associated with a very small statistically significant increase in exercise behaviours and more positive attitudes towards physical activity in the short term. The improvements observed two weeks post-event are consistent with previous findings indicating that structured, time-limited initiatives can temporarily enhance engagement in physical activity (Brown et al., 2006). However, no significant changes were sustained to three-month follow-up, suggesting that the Push-Up Challenge may be insufficient to produce lasting change in exercise habits and the potential need for ongoing support or reinforcement strategies to maintain improvements in exercise behaviours and attitudes over time.

### Resilience

Analysis revealed significant increases in resilience post-event, though these gains were not sustained at the three-month follow-up. Participation in events like the Push-Up Challenge may promote resilience by increasing physical activity, which can improve self-regulation and buffer stress (Belcher et al., 2021). However, while such mechanisms may lead to short-term improvements, the lack of longer-term effects could reflect the limited duration of the Push-Up Challenge. Other programs have similarly reported that initial gains in resilience tend to decline over time without ongoing engagement (Liossis et al., 2009). Notably, as exercise behaviours were not maintained beyond the immediate post-event period, any resilience benefits that depended on continued physical activity may also diminish accordingly.

### Social Connection

Analysis found a significant improvement in social connection post-event, although the effect size was small. However, no significant improvements in social connection were observed at three-month follow-up. This may reflect reduced opportunities for social interaction once the event concluded, particularly for those participating in a team. These findings are in line with literature suggesting that the relationship between exercise and positive changes in mental health and wellbeing may, in part, be mediated by opportunities for social connection created through exercise (Mason & Holt, 2012).

### Mental Health Help-Seeking and Self-Care Behaviours

Participants in this study were over twice as likely to seek help for a mental health problem and over three times as likely to adopt self-care strategies at both post-event and three-month follow-up, representing substantial shifts in these behaviours. While some previous mental health awareness interventions have successfully influenced participants’ intentions to seek help for mental health concerns (Lindow et al., 2020), such interventions targeting the general public do not always translate into actual increases in help-seeking behaviour or adoption of self-care strategies (Xu et al., 2018). In contrast, the reported increase in both help-seeking and self-care behaviours observed in this evaluation highlights a key strength of the Push-Up Challenge as an intervention that may be fostering tangible behaviour change in its participants. One possible explanation for this effect is that the intervention encouraged participants to share its mental health-related information on social media during the event, potentially amplifying the campaign’s messages on help-seeking, mental health promotion, and available services among networks of participants.

Research into the effectiveness of social media-based campaigns has identified key enablers of engagement, including the sharing of video content over static images and trust in the organisation overseeing the campaign (Draganidis et al., 2024). However, while higher engagement levels may enhance message reach but do not necessarily lead to behaviour change. Although the daily mental health-related information provided by the Push-Up Challenge, and the ways in which participants share this content within their social networks are not explicitly grounded in a formal behaviour change theory, the campaign’s effectiveness in influencing participant behaviour may be attributed to its alignment with key aspects of theories related to mental health help-seeking, such as addressing participants’ attitudes, social norms, and perceived opportunities, skills, and resources (Adams et al., 2022).

### Engaging Men in Mental Health Promotion Interventions

Characteristics of the study sample provide important context for understanding the impact of this intervention. Most participants in this evaluation were male, reflecting the population that took part in the Push-Up Challenge event itself (64.8% vs. 67.2% male in the total cohort). Previous Australian research has identified effective strategies for engaging men in mental health promotion activities, including campaigns that emphasise action-oriented behaviours such as helping others, and that avoid explicitly framing the campaign around participants’ own mental health (Sharp et al., 2022). The Push-Up Challenge’s emphasis on “mental fitness” and exercise, rather than directly focusing on mental health, aligns with these findings. Its core aim of supporting the community by raising awareness and funds for mental health and suicide prevention appears to have successfully engaged a substantial proportion of male participants. The effectiveness of this messaging is consistent with broader research indicating that men tend to prefer activity-based interventions over talk-based approaches for mental health promotion (Kielan et al., 2020).

### Strengths and Limitations

This study is the first independent evaluation of The Push-Up Challenge. Key strengths include the recruitment of a large sample and the use of mixed-effects modelling. However, as a pre-post study without a control group, it cannot establish causation and observed changes may be influenced or caused by external factors. This design is also susceptible to regression to the mean and expectancy effects, which may have contributed to observed changes. High attrition rates further limit generalisability and may have introduced bias, as participants who remained engaged at follow-up could have been more motivated to continue or experienced better outcomes, leading to overestimation of intervention effects. These biases may have been amplified in the post-hoc analyses, which relied on data from optional additional questions and were thus subject to greater missingness and self-selection effects. While analyses attempted to mitigate such bias by controlling for factors associated with missingness, limitations remain.

Measurement-related issues should also be considered in interpretation. The shorter survey instruments used to assess depression and resilience showed weaker internal consistency, reducing confidence in their measurement of the intended constructs. Some outcomes were assessed using single-item scales, limiting the reliability and validity. Additionally, reliance on self-report measures may have introduced bias due to social desirability or inaccurate self-assessment. Data were not collected on socioeconomic status, income, or cultural background, restricting evaluation of the sample’s representativeness and limiting generalisability to the broader population.

### Future Directions

Further research could investigate the relationship between changes in physical activity and wellbeing, and whether this relationship is influenced by social connection or taking part in a group, as suggested by previous studies (Chekroud et al., 2018). Such analysis would provide greater insight into the mechanisms through which The Push-Up Challenge achieves its outcomes, particularly if designed to reduce attrition and using controlled, longitudinal designs. While the intervention was associated with improvements in physical activity attitudes and behaviours immediately following the event, these gains did not persist at three-month follow-up. This suggests a need to explore strategies for enhancing the sustainability of behaviour change, such as integrating follow-up components that encourage ongoing physical activity or reinforcing social connections developed during The Push-Up Challenge. Additionally, this evaluation did not examine the behaviour change theories underlying this intervention. A deeper exploration of the mechanisms and theoretical foundations driving the intervention’s outcomes could identify which aspects of The Push-Up Challenge are the most effective. Strengthening these theoretical underpinnings has the potential to improve participant outcomes, both broadly and within specific groups, such as individuals facing mental health challenges. This aligns with evidence from systematic reviews of internet-based health promotion interventions, which suggest that integrating behaviour change theories leads to more effective outcomes (Webb et al., 2010).

## Conclusion

The findings of this evaluation suggest that the Push-Up Challenge has the potential to contribute positively to various aspects of mental and physical wellbeing, albeit with limitations in the magnitude and longevity of its effects. It was also particularly effective in engaging men; a group often harder to engage in mental health interventions. However, the modest and sometimes brief nature of the observed changes indicates a need for strategies that extend the intervention’s effects beyond the duration of the event. In particular, the transience of improvements in resilience, social connection, and physical activity behaviours suggest where improvements could be targeted in supporting long-term engagement and sustained behaviour change. The Push-Up Challenge’s multicomponent model, which blends exercise with mental health literacy and awareness, remains a promising foundation. Future iterations may benefit from embedding mechanisms that promote continued participation in group-based exercise activities or reinforce key messages and behaviours after the event concludes.

## References

[CR1] ABS (2023). *Causes of Death, Australia*. Australian Bureau of Statistics. https://www.abs.gov.au/statistics/health/causes-death/causes-death-australia/latest-release

[CR2] ABS (2020). *National Study of Mental Health and Wellbeing*. Australian Bureau of Statistics. https://www.abs.gov.au/statistics/health/mental-health/national-study-mental-health-and-wellbeing/latest-release

[CR3] Adams, C., Gringart, E., & Strobel, N. (2022). Explaining adults’ mental health help-seeking through the lens of the theory of planned behavior: A scoping review. *Systematic Reviews*, *11*(1), 160. 10.1186/s13643-022-02034-y 35945633 10.1186/s13643-022-02034-y PMC9361557

[CR4] AIHW (2024). *Burden of disease—Mental health*. Australian Institute of Health and Welfare. https://www.aihw.gov.au/mental-health/snapshots/burden-of-disease

[CR5] Anwar-McHenry, J., Donovan, R. J., Jalleh, G., & Laws, A. (2012). Impact evaluation of the Act‐Belong‐Commit mental health promotion campaign. *Journal of Public Mental Health,**11*(4), 186–194. 10.1108/17465721211289365

[CR6] Australian Government Department of Health and Aged Care (2021). *Physical Activity and Exercise Guidelines for All Australians*. Australian Government. https://www.health.gov.au/topics/physical-activity-and-exercise/physical-activity-and-exercise-guidelines-for-all-australians

[CR7] Bauman, A., McNeil, N., Nicholson, M., O’Halloran, P., Seal, E., Randle, E., & Stukas, A. (2023). Impact of the first year of the this Girl can physical activity and sport mass media campaign in Australia. *Bmc Public Health*, *23*(1), 333. 10.1186/s12889-023-15091-2 36793043 10.1186/s12889-023-15091-2 PMC9930268

[CR8] Belcher, B. R., Zink, J., Azad, A., Campbell, C. E., Chakravartti, S. P., & Herting, M. M. (2021). The roles of physical activity, exercise, and fitness in promoting resilience during adolescence: Effects on mental well-being and brain development. *Biological Psychiatry: Cognitive Neuroscience and Neuroimaging,**6*(2), 225–237. 10.1016/j.bpsc.2020.08.005 33067166 10.1016/j.bpsc.2020.08.005 PMC7878276

[CR9] Brown, W., Mummery, W., Eakin, E., & Schofield, G. (2006). 10,000 Steps rockhampton: Evaluation of a whole community approach to improving population levels of physical activity. *Journal of Physical Activity & Health*. 10.1201/b15877-2

[CR10] Chekroud, S. R., Gueorguieva, R., Zheutlin, A. B., Paulus, M., Krumholz, H. M., Krystal, J. H., & Chekroud, A. M. (2018). Association between physical exercise and mental health in 1·2 million individuals in the USA between 2011 and 2015: A cross-sectional study. *The Lancet Psychiatry*, *5*(9), 739–746. 10.1016/S2215-0366(18)30227-X 30099000 10.1016/S2215-0366(18)30227-X

[CR11] Des Jarlais, D. C., Lyles, C., Crepaz, N., & Group, T. (2004). Improving the reporting quality of nonrandomized evaluations of behavioral and public health interventions: The TREND statement. *American Journal of Public Health*, *94*(3), 361–366. 14998794 10.2105/ajph.94.3.361 PMC1448256

[CR12] Draganidis, A., Fernando, A. N., West, M. L., & Sharp, G. (2024). Social media delivered mental health campaigns and public service announcements: A systematic literature review of public engagement and help-seeking behaviours. *Social Science & Medicine*, *359*, 117231. 10.1016/j.socscimed.2024.117231 39278158 10.1016/j.socscimed.2024.117231

[CR13] Godin, G., & Shephard, R. (1985). A simple method to assess exercise behavior in the community. *Can J Appl Sport Sci,**10*(3), 141–146. 4053261

[CR14] Golaszewski, N., LaCroix, A., Hooker, S., & Bartholomew, J. (2022). Group exercise membership is associated with forms of social support, exercise identity, and amount of physical activity. *International Journal of Sport and Exercise Psychology*, *20*(2), 630–643. 10.1080/1612197x.2021.1891121 35494549 10.1080/1612197x.2021.1891121 PMC9053316

[CR15] Jorm, A. F., Nakane, Y., Christensen, H., Yoshioka, K., Griffiths, K. M., & Wata, Y. (2005). Public beliefs about treatment and outcome of mental disorders: A comparison of Australia and Japan. *BMC Medicine*, *3*(1), 12. 10.1186/1741-7015-3-12 16004615 10.1186/1741-7015-3-12 PMC1177951

[CR16] Kielan, A. J., Stradomska, M., Jaworski, M., Mosiołek, A., Chodkiewicz, J., Święcicki, Ł., & Walewska-Zielecka, B. (2020). Promotion of men’s mental health. *Psychiatria*, *17*(4), 212–215. 10.5603/PSYCH.2020.0035

[CR17] Kroenke, K., Spitzer, R. L., & Williams, J. B. W. (2001). The PHQ-9. *Journal of General Internal Medicine*, *16*(9), 606–613. 10.1046/j.1525-1497.2001.016009606.x 11556941 10.1046/j.1525-1497.2001.016009606.x PMC1495268

[CR18] Kvam, S., Kleppe, C. L., Nordhus, I. H., & Hovland, A. (2016). Exercise as a treatment for depression: A meta-analysis. *Journal of Affective Disorders*, *202*, 67–86. 10.1016/j.jad.2016.03.063 27253219 10.1016/j.jad.2016.03.063

[CR19] Lindow, J. C., Hughes, J. L., South, C., Minhajuddin, A., Gutierrez, L., Bannister, E., Trivedi, M. H., & Byerly, M. J. (2020). The youth aware of mental health intervention: Impact on help seeking, mental health knowledge, and stigma in U.S. adolescents. *Journal of Adolescent Health,**67*(1), 101–107. 10.1016/j.jadohealth.2020.01.006 10.1016/j.jadohealth.2020.01.006 PMC7311230 32115325

[CR20] Liossis, P. L., Shochet, I. M., Millear, P. M., & Biggs, H. (2009). The promoting adult resilience (PAR) program: The effectiveness of the second, shorter pilot of a workplace prevention program. *Behaviour Change*, *26*(2), 97–112. 10.1375/bech.26.2.97

[CR21] Livingston, J. D., Tugwell, A., Korf-Uzan, K., Cianfrone, M., & Coniglio, C. (2013). Evaluation of a campaign to improve awareness and attitudes of young people towards mental health issues. *Social Psychiatry and Psychiatric Epidemiology*, *48*(6), 965–973. 10.1007/s00127-012-0617-3 23124481 10.1007/s00127-012-0617-3

[CR22] Löwe, B., Kroenke, K., & Gräfe, K. (2005). Detecting and monitoring depression with a two-item questionnaire (PHQ-2). *Journal of Psychosomatic Research*, *58*(2), 163–171. 10.1016/j.jpsychores.2004.09.006 15820844 10.1016/j.jpsychores.2004.09.006

[CR23] Mahindru, A., Patil, P., & Agrawal, V. (2023). Role of physical activity on mental health and well-being: A review. *Cureus,**15*(1), e33475. 10.7759/cureus.33475 36756008 10.7759/cureus.33475 PMC9902068

[CR24] Malone, G. P., Pillow, D. R., & Osman, A. (2012). The general belongingness scale (GBS): Assessing achieved belongingness. *Personality and Individual Differences*, *52*(3), 311–316. 10.1016/j.paid.2011.10.027

[CR25] Marinelli, R., Parker, A. G., Levinger, I., Bourke, M., Patten, R., & Woessner, M. N. (2024). Resistance training and combined resistance and aerobic training as a treatment of depression and anxiety symptoms in young people: A systematic review and meta-analysis. *Early Intervention in Psychiatry,**18*(8), 585–598. 10.1111/eip.13528 38710640 10.1111/eip.13528

[CR26] Mason, O. J., & Holt, R. (2012). Mental health and physical activity interventions: A review of the qualitative literature. *Journal of Mental Health*, *21*(3), 274–284. 10.3109/09638237.2011.648344 22533784 10.3109/09638237.2011.648344

[CR27] Mok, K., Donovan, R., Hocking, B., Maher, B., Lewis, R., & Pirkis, J. (2016). Stimulating community action for suicide prevention: Findings on the effectiveness of the Australian R U OK? campaign. *International Journal of Mental Health Promotion,**18*(4), 213–221. 10.1080/14623730.2016.1209423

[CR28] Niederkrotenthaler, T., & Till, B. (2020). Effects of suicide awareness materials on individuals with recent suicidal ideation or attempt: Online randomised controlled trial. *The British Journal of Psychiatry,**217*(6), 693–700. 10.1192/bjp.2019.259 31843026 10.1192/bjp.2019.259

[CR29] Pascoe, M., Bailey, A. P., Craike, M., Carter, T., Patten, R., Stepto, N., & Parker, A. (2020). Physical activity and exercise in youth mental health promotion: A scoping review. *BMJ Open Sport & Exercise Medicine*. 10.1136/bmjsem-2019-000677 10.1136/bmjsem-2019-000677 PMC7010991 32095272

[CR30] Rose, G., Khaw, K. T., & Marmot, M. G. (2008). *Rose’s Strategy of Preventive Medicine*. Oxford University Press.

[CR31] Rosenbaum, S., & Sherrington, C. (2011). Is exercise effective in promoting mental well-being in older age? A systematic review. *British Journal of Sports Medicine*, *45*(13), 1079–1080. 10.1136/bjsports-2011-090466 21926078 10.1136/bjsports-2011-090466

[CR32] Ross, A. M., & Bassilios, B. (2019). Australian R U OK?day campaign: Improving helping beliefs, intentions and behaviours. *International Journal of Mental Health Systems,**13*(1), 61. 10.1186/s13033-019-0317-4 31534474 10.1186/s13033-019-0317-4 PMC6744695

[CR33] Sharp, P., Bottorff, J. L., Rice, S., Oliffe, J. L., Schulenkorf, N., Impellizzeri, F., & Caperchione, C. M. (2022). People say men don’t talk, well that’s bullshit: A focus group study exploring challenges and opportunities for men’s mental health promotion. *PLoS One,**17*(1), Article e0261997. 10.1371/journal.pone.0261997 35061764 10.1371/journal.pone.0261997 PMC8782463

[CR34] Spitzer, R. L., Kroenke, K., Williams, J. B. W., & Löwe, B. (2006). A brief measure for assessing generalized anxiety disorder: The GAD-7. *Archives of Internal Medicine*, *166*(10), 1092–1097. 10.1001/archinte.166.10.1092 16717171 10.1001/archinte.166.10.1092

[CR35] Stubbs, B., Vancampfort, D., Rosenbaum, S., Firth, J., Cosco, T., Veronese, N., Salum, G. A., & Schuch, F. B. (2017). An examination of the anxiolytic effects of exercise for people with anxiety and stress-related disorders: A meta-analysis. *Psychiatry Research*, *249*, 102–108. 10.1016/j.psychres.2016.12.020 28088704 10.1016/j.psychres.2016.12.020

[CR36] Tam, M. T., Wu, J. M., Zhang, C. C., Pawliuk, C., & Robillard, J. M. (2024). A systematic review of the impacts of media mental health awareness campaigns on young people. *Health Promotion Practice*, *25*(5), 907–920. 10.1177/15248399241232646 38468568 10.1177/15248399241232646 PMC11370183

[CR37] Tennant, R., Hiller, L., Fishwick, R., Platt, S., Joseph, S., Weich, S., Parkinson, J., Secker, J., & Stewart-Brown, S. (2007). The Warwick-Edinburgh mental well-being scale (WEMWBS): Development and UK validation. *Health and Quality of Life Outcomes,**5*(1), 63. 10.1186/1477-7525-5-63 18042300 10.1186/1477-7525-5-63 PMC2222612

[CR38] Vaishnavi, S., Connor, K., & Davidson, J. R. T. (2007). An abbreviated version of the Connor-Davidson resilience scale (CD-RISC), the CD-RISC2: Psychometric properties and applications in psychopharmacological trials. *Psychiatry Research,**152*(2), 293–297. 10.1016/j.psychres.2007.01.006 17459488 10.1016/j.psychres.2007.01.006 PMC2041449

[CR39] Webb, T., Joseph, J., Yardley, L., & Michie, S. (2010). Using the internet to promote health behavior change: A systematic review and Meta-analysis of the impact of theoretical basis, use of behavior change techniques, and mode of delivery on efficacy. *Journal of Medical Internet Research*, *12*(1), e1376. 10.2196/jmir.1376 10.2196/jmir.1376 PMC2836773 20164043

[CR40] Wheatley, C., Glogowska, M., Stathi, A., Sexton, C., Johansen-Berg, H., & Mackay, C. (2021). Exploring the public health potential of RED january, a social media campaign supporting physical activity in the community for mental health: A qualitative study. *Mental Health and Physical Activity*, *21*, 100429. 10.1016/j.mhpa.2021.100429 35154380 10.1016/j.mhpa.2021.100429 PMC7612346

[CR41] Xu, Z., Huang, F., Kösters, M., Staiger, T., Becker, T., Thornicroft, G., & Rüsch, N. (2018). Effectiveness of interventions to promote help-seeking for mental health problems: Systematic review and meta-analysis. *Psychological Medicine*, *48*(16), 2658–2667. 10.1017/S0033291718001265 29852885 10.1017/S0033291718001265

